# Real-World Yield of the RAISE Score for Cardiac Amyloidosis Screening in Patients Undergoing Transcatheter Aortic Valve Replacement

**DOI:** 10.1016/j.cjco.2026.06.017

**Published:** 2026-08-01

**Authors:** Michele Bertelli, Robert J.H. Miller, Anna Bizios, Debra Bosley, Annette Gourlay, Lyndsay Litwin, Ayaaz Sachedina, Daniel Holloway, Nishant Sharma, Nowell M. Fine

**Affiliations:** Department of Cardiac Sciences, University of Calgary, Calgary, Alberta, Canada

**Keywords:** cardiac amyloidosis, transthyretin amyloidosis, pyrophosphate scan, aortic stenosis, transcatheter aortic valve replacement, RAISE score

Cardiac amyloidosis (CA) is a progressive infiltrative cardiomyopathy associated with high mortality when untreated. Wild-type transthyretin amyloidosis cardiomyopathy (ATTR-CM) is the most common CA subtype and is frequently associated with aortic stenosis (AS). Recent observational studies estimate the prevalence of ATTR-CM among patients with severe AS to be 5%29%,^1^ derived mostly from transcatheter aortic valve replacement (TAVR) referral populations. With the availability of CA disease-modifying therapies, early recognition of CA among AS patients is critical to improving survival. However, correct recognition is challenging due to overlapping clinical and imaging features, leading to growing efforts to stratify CA risk among AS patients.^2^

The RAISE score (for remodelling, age, injury, systemic, electrical) is a validated risk score predicting the likelihood of CA among patients referred for TAVR.^2^ The score incorporates clinical, laboratory, electrocardiographic, and echocardiographic data across 5 domains (cardiac remodelling, age, myocardial injury, systemic manifestation, and electrocardiographic changes), with a score ≥ 3 demonstrating 72.3% sensitivity and 83.6% specificity for CA detection.^2^ The diagnostic performance of the score has been evaluated in patients undergoing surgical aortic valve replacement, demonstrating lower sensitivity than originally reported.^3^ In addition, reporting of real-world applications of the RAISE score has been limited,^4^ with a recent study demonstrating a prevalence of CA with scores ≥ 3 as low as 4.5%,^5^ raising questions regarding the utility of its routine application in clinical practice. This study was a retrospective analysis of a clinical pilot initiative conducted at a Canadian amyloidosis referral centre using the RAISE score to guide CA screening in TAVR recipients.

A total of 262 consecutive patients who underwent TAVR for severe AS at the University of Calgary (Calgary, Alberta, Canada) between May 2025 and May 2026 were included. For each patient, the RAISE score was calculated, with the following distribution observed: score 0 = 16.8%; score 1 = 50.0%; score 2 = 16.0%; score 3 = 6.1%; score 4 = 7.3%; score 5 = 2.7%; score 6 = 0.8%; and score 7 = 0.4%. CA testing, namely serum and urine monoclonal protein screening to exclude light chain (AL) CA, and if negative followed by ^99m^technetium-pyrophosphate (Tc-99m-PYP) nuclear scintigraphy, was offered to patients with a score ≥ 3. Forty-five patients (17%) met this criterion and underwent testing (Fig. 1). Twenty-three patients did not undergo CA testing despite meeting the cutoff. This group included 13 patients who were not offered testing after clinical and imaging review by specialized amyloidosis cardiologists (N.M.F. and R.J.H.M.; 12 had imaging features inconsistent with ATTR-CM, and 1 had end-stage disease), and 10 patients who were offered testing but declined further workup. Of the 22 who underwent CA testing, none were diagnosed with AL CA, and 6 (27.3%) were diagnosed with ATTR-CM (2.3% of the total screening population). All 6 patients had wild-type (age-related) ATTR-CM subtype, based on negative testing for a transthyretin gene mutation, with 5 diagnosed by Tc-99m-PYP and 1 diagnosed by endomyocardial biopsy following an inconclusive scintigraphy result. Their RAISE score distribution was as follows: score 3 = 2 patients (12.5%); score 4 = 3 patients (15.8%); and score 5 = 1 patient (14.3%). Five of 6 ATTR-CM patients were initiated on tafamidis disease-modifying therapy, whereas 1 was not, due to having end-stage disease. All patients initiated on tafamidis therapy had symptomatic ATTR-CM and met eligibility criteria for treatment.

**Figure 1:**
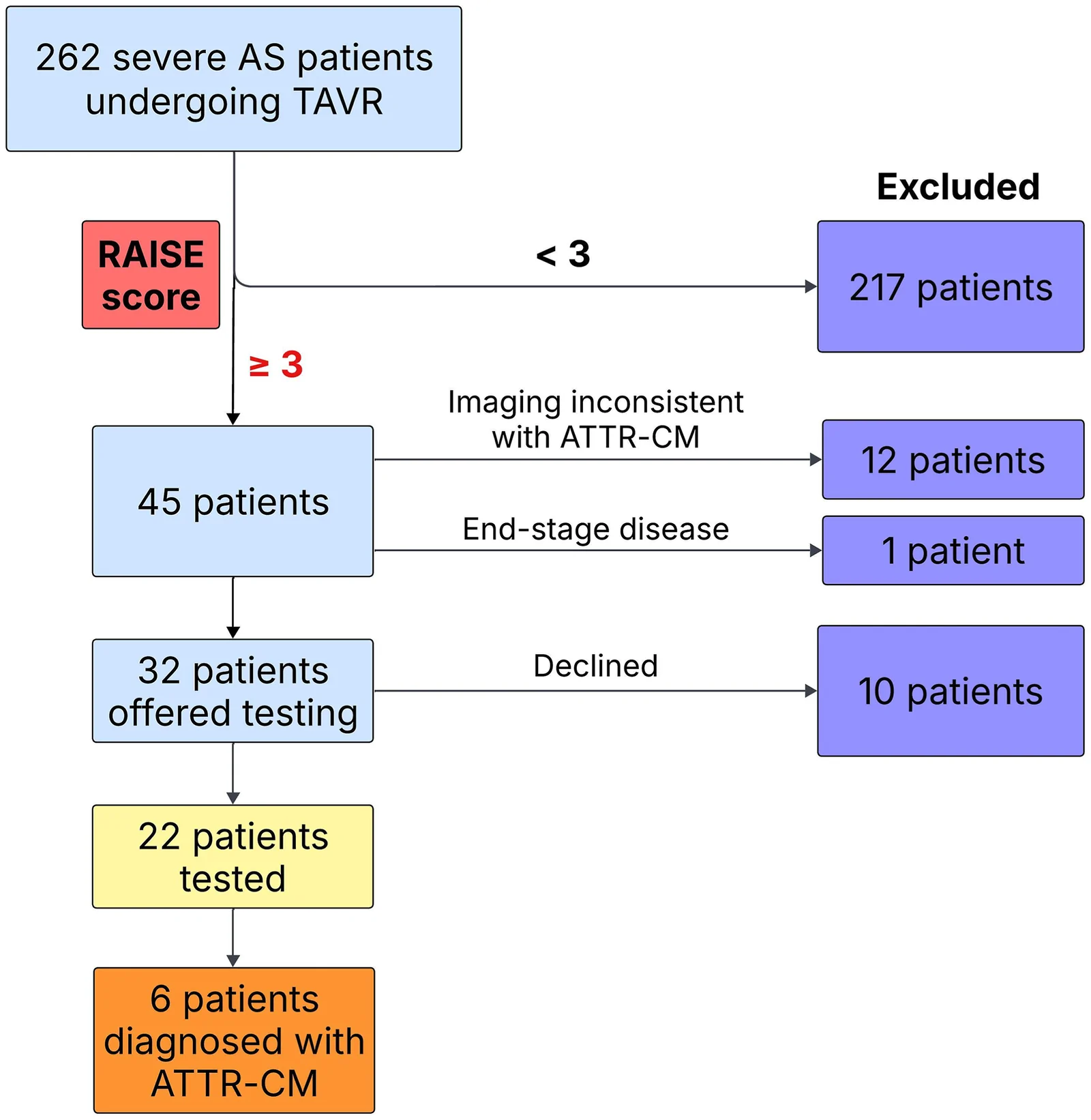
Flowchart demonstrating screening and testing for cardiac amyloidosis (CA) in aortic stenosis (AS) patients referred for transcatheter aortic valve replacement (TAVR). ATTR-CM: transthyretin amyloidosis cardiomyopathy; RAISE, remodelling, age, injury, systemic, electrical.

In this clinical pilot study, we assessed the feasibility and yield of applying the RAISE score as a screening tool to direct CA workup in AS patients undergoing TAVR over a 12-month period. Although our study demonstrates the feasibility of incorporating this score into a multidisciplinary clinical practice model, it also raises significant questions about its utility in real-world practice. Approximately 17% of those screened met criteria for further testing, and 8% underwent such testing (with the remainder either declining or being determined to not warrant further testing after a clinical and imaging review by an amyloidosis specialist). Overall, 2% of patients were diagnosed with ATTR-CM. This prevalence is lower than that in the original RAISE study (for remodelling, age, injury, systemic, electrical) which identified a CA prevalence of approximately 12% of those screened among a TAVR referral population.^2^ Although 12 patients who met the score cutoff were not offered testing, this was predominantly due to the absence of imaging features consistent with ATTR-CM—that is, they had normal left ventricular wall thickness. The prevalence of ATTR-CM with normal left ventricular wall thickness is known to be very low,^6^ and therefore proceeding with further diagnostic testing was felt to be clinically inappropriate, although this lack of testing represents a limitation of our analysis. Our study is also limited by its sample size and single-centre, retrospective design. In addition, detailed comparative data for patients who did and did not undergo further testing for ATTR-CM after screening were not available. Our exploratory clinical pilot study using the RAISE score for CA screening among TAVR patients demonstrated a very low diagnostic yield. Larger prospective studies are therefore needed to determine the optimal approach to CA screening in this population, and whether this strategy is cost-effective and leads to improvements in long-term outcomes.

## Patient Consent

The authors confirm that patient consent forms have been obtained for this article.

## Funding Sources

The authors have no funding sources to declare.

## Disclosures

R.J.J.M. reports receiving research support from Alberta Innovates and consulting fees from Alnylam and Bayer. A.B. reports receiving honoraria from Edwards Lifesciences. A.S. reports a consultancy with Boston Scientific. N.M. Fine reports receiving consulting/speaking honoraria and research support from AstraZeneca/Alexion, Alnylam, BidgeBio, Novo Nordisk and Pfizer. All the other authors have no conflicts of interest to disclose.
